# The Health of Woman as Compared to That of Man, Especially in Relation to Marriage

**Published:** 1884-06-20

**Authors:** George Hamilton

**Affiliations:** Philadelphia


### THE HEALTH OF WOMAN AS COMPARED TO THAT OF MAN, ESPECIALLY IN RELATION TO MARRIAGE.

By GEORGE HAMILTON, M. D., OF PHILADELPHIA.

This subject has, for some time past, received a good deal of attention, and, during the few past years, it has been made to assume increased importance, not only in medical lectures and writings, but also in the transactions of associations established for the advancement of hygiene. As a rule, it will be found that the status of woman, in regard to health, is represented to be much lower than that of man. In this point of view it is not surprising that, from the time she has arrived at marriageable age, attention is chiefly given to this period of her life, and expressions of regret are often heard that so many are ill-prepared for the duties and exigencies of married life. But while this unfavorable view is in many cases justified, is it not far more frequently entertained than a due consideration of the facts pertaining to this subject will warrant? Is there anything at the moment of birth, or for years after, indicative of a more feeble constitution, or greater proclivity to disease, in the female than in the male, so that doubts might arise as to her capacity to occupy the future position assigned her by nature? On the contrary, statistics show that more male than female children are still-born, and also that more males from birth to the fifth year perish than females, chiefly owing to their greater disposition to disease of the brain. As the female approaches womanhood, and for some years after, especially if she marry very young, the chances for health and longevity are to some degree diminished. Taking the entire period of childbearing, statistics are not in accord as to the comparative mortality; but beyond that period the chances for health and length of days turn strongly in favor of the woman. But this prolonged existence may readily be explained in the fact that not very many women are addicted to the use or abuse of intoxicating liquors—an abuse of a character, as all statistics prove, to cause in men an enormous amount of physical and mental disease. To agencies of another kind are often attributed the unfitness of very many females for the duties and trials of wedded existence. Lecturers and writers upon this subject have, as it were by common accord, placed in this class many of the daughters of wealth and fashion. In this class, as in every other, may be found those who, by inheritance or otherwise, possess feeble constitutions, and who in matrimony may not only incur risk of further injury to health, but whose offspring may have constitutions similar to those of the mothers. Another class, having good health and constitution, may, through a constant, protracted course of fashionable dissipation, destroy or greatly impair both, and may thus, so far as matrimony is concerned, be placed in the category alluded to. The number in both classes is, the writer has reason to believe, comparatively small; so small, indeed, as not to justify a general charge against this condition of society, as an appeal to facts may prove.

Let us, for instance, stroll, at certain hours of the day, through that portion of the city which, par excellence, may be regarded as one of the most fashionable and wealthy, having as its centre Rittenhouse Square, and what will at once gratify and attract our attention so much as the numerous groups of children in and about the square, of every age, from the infant of a few months or a year, up to children of, perhaps, eight or ten years? Infants, too young to walk, are being trundled about in all directions, their plump forms, rosy complexions, bright eyes and cheerful looks, giving visible evidence of their excellent health. Others of suitable age are, by direction of intelligent parents, instructed to avoid the coach, and thus bring the muscles of the limbs into action, and thereby increase the appetite and power of digestion, so as to improve the strength and tone of the whole system. When old enough, the children (girls as well as boys) are provided with a variety of implements used in various amusing and healthful games, or with one or other of the many machines now in vogue for rapid and pleasant locomotion. But again, the residences of these children are nearly always large, admitting perfect ventilation, and provided with conveniences that conduce, in no small degree, to the comfort and well-being of the occupants. Another point, greatly advantageous to daughters, from childhood to womanhood, is that, as a rule, the parents are educated and refined, and are, therefore, the better able to discover and carry into effect all that pertains to the physical, intellectual and moral welfare of their children, especially as regards the daughters

Thus far, it may be said, very good; but, arrived at womanhood, then comes the trial, by entering upon a round of fashionable dissipation that too often attends the social status in view, especially when no incumbent occupation or duties interpose an obstacle. But there is another side to this picture, in the fact that only a part of the time is thus employed, and a very large number of these devotees of fashion spend much of their time in riding in the open air, or, like the ladies of England, in promenading—the best of all exercises. Again, as soon as the season arrives for leaving the city, a very large majority close their houses, repair to their summer homes, or to the almost innumerable places of resort by the seaside, or to situations (in rural sections) noted for their salubrity. Here their enjoyments are, in a great measure, of an active kind, and are thus productive of increased health and strength.

That there are other, and quite opposite, conditions of society, in which the females are incomparably less healthy than those in question, is beyond doubt—and have far less qualification for matrimony or longevity.

But what are the actual qualifications of man for matrimony, as compared with woman? If the statistics of some writers be correct—that the mortality of woman, during the whole period of child-bearing, is about the same as that of man for the same period—then the conclusion is inevitable that some deleterious agents are in operation upon man; for women must surely encounter certain risks peculiar to pregnancy and accouchement.

As intimated above, the use, or rather abuse, of intoxicating liquors, is so common with men that probably ten of them suffer mentally and physically from this cause for one woman. But this is not all; for, as is well known, the stimulus of alcohol, in any form, is too often the stimulus of every evil passion; and as men generally defer marriage to a later period than women, so are they more apt to become the victims of dissipation of every kind, and consequent disease or debilitation. This condition exists in every class of society; and where the pecuniary condition is abundant, the temptation and inducement are thereby increased.

From this statement, may it not very often happen that favorable qualifications for matrimony are deficient on the side of the man rather than of the woman?

The writer is aware that this view may not accord with that of the gynæcologist or the special accoucheur; but would not the large amount ot this special practice induce those engaged in it to imagine that such cases are far more numerous than the general practitioner could admit?

In the "Report of the Medical Association of Missouri for 1883" may be found a well-written and elaborate paper upon this subject. After making due allowance for such cases in men—accidents, dissipation or other agencies—and allowing in like manner for such influences as may at certain periods affect females unfavorably, Prof. Schenck, the author of the paper, seems inclined to the opinion that woman has the greater vitality, and that under equal conditions she has a better prospect of length of days. This opinion, granting that equal conditions were possible, is well-founded; but while man, in general, has greater muscular power, that is no evidence of greater, or even equal vitality; for it not unfrequently happens that neither the man nor the woman of large bone and muscle has the hardihood and endurance of persons of moderate conformation. But was it not absolutely necessary, in view of the destined life of the woman, that an all-wise Providence should endow her with an amount of vital endurance commensurate with the tax upon her constitution that pregnancy, child-birth, lactation and the care of her offspring would impose? In regard to ability to support attacks of fever or other acute disease, the writer has generally found the advantage to be on the side of the woman; and the accoucheur and the gynecologist, in particular, could not fail to have observed the frequent recuperation of females after extensive surgical operations and immense losses of blood occasioned thereby, or as occur in cases of abortion and accouchement.—*Med. and Surg. Reporter.*

